# Massachusetts Dental Society

**Published:** 1865

**Authors:** Thos. H. Chandler


### MASSACHUSETTS DENTAL SOCIETY.

The second annual meeting of the Massachusetts Dental Society was held in Boston on May 18th. The usual reports were read and accepted, showing the society to be in all respects in a flourishing condition. The number of its members has more than doubled during the year. The library and cabinet has made an excellent beginning, and the treasury shows a large balance in hand. The charter, granted by the Massachusetts Legislature at its last session, was accepted, and the society is now an incorporated body. The following named gentlemen were chosen officers for the ensuing year, viz:

Dr. N. C. Keep, M. D., *President.*
" E. G. Leach, *Vice-President.*
" T. H. Chandler, *Recording Secretary.*
" E. C. Rolfe, M. D., *Corresponding Secretary.*
" S. J. McDougall, M. D., *Treasurer.*
" E. N. Harris, D. D. S., *Librarian.*
" A. Lawrence, M. D.
" J. T. Codman,
" H. F. Bishop, D. D. S.
" J. B. Hitchcock, M. D.
" T. F. Ham.
} *Executive Committee.*

The number of delegates to attend the American Dental Association to which the society is entitled being ten, the following named gentlemen were unanimously chosen, viz: Drs. Wetherbee, Lawrence, Keep, Leach, Bishop, Harris, Gerry, Shepard, Rolfe and Hitchcock.

Dr. I. J. Wetherbee was appointed to deliver the address at our next anniversary meeting with Dr. E. C. Rolfe as his alternate.

The following preamble and resolutions offered by Dr. Wetherbee, being substantially the same, as were adopted by the American Dental Association and American Dental Convention at their meetings the last year, were unanimonsly passed, viz:

WHEREAS, In the opinion of the Massachusetts Dental Society, not less than two years pupilage in the office of a competent dentist, and attendance upon two full courses of lectures in a dental college, will qualify an individual to practice dentistry properly; therefore,

*Resolved*, That practitioners of dentistry be requested not to receive into their offices students for a less term than two years, and under no consideration unless they agree to attend lectures in and be graduated from a dental college, before entering upon the practice of the profession.

*Resolved*, That it is the duty as well as the interest of the people to require of all who hereafter enter on the practice of dentistry that they shall have received in course a diploma of graduation from a dental college as the first requisite for public confidence and patronage.

*Resolved*, That these resolutions be published.

The President, Dr. N. C. Keep, then delivered an earnest and eloquent address, which was listened to with interested attention, eliciting repeated bursts of applause; at the conclusion it was voted "that the thanks of the Society be tendered to our honored President for the kind words of advice and encouragement he has given us, and that a copy of his address be requested for preservation in our archives and for publication." It was also voted "that the annual address be published in pamphlet form for the benefit of the members;" also, "that a condensed report of the proceedings of this meeting be published in the dental journals, and in such of the city journals as the Secretary may see fit."

At the conclusion of the business transactions the members adjourued to the Tremont House where they partook of an excellent dinner and enjoyed therewith the eloquent speeches of the gifted among their number.

THOS. H. CHANDLER, *Secretary.*

